# Expression of GABA receptor subunits in the hippocampus and thalamus after experimental traumatic brain injury

**DOI:** 10.1016/j.neuropharm.2014.08.023

**Published:** 2015-01

**Authors:** Meinrad Drexel, Noora Puhakka, Elke Kirchmair, Heide Hörtnagl, Asla Pitkänen, Günther Sperk

**Affiliations:** a Department of Pharmacology, Innsbruck Medical University, 6020 Innsbruck, Austria; b Department of Neurobiology, A. I. Virtanen Institute for Molecular Science, University of Eastern Finland, PO Box 1627, FI-70211 Kuopio, Finland; c Department of Neurology, Kuopio University Hospital, PO Box 1777, FI-70211 Kuopio, Finland

**Keywords:** Epilepsy, GABA, Lateral fluid-percussion injury, Neurosteroid

## Abstract

Traumatic brain injury is a major cause of death and disability worldwide and often associated with post-traumatic epilepsy. We recently demonstrated that TBI induces acquired GABA_A_ receptors channelopathy that associates with hyperexcitability in granule cell layer (GCL). We now assessed the expression of GABA_A_ and GABA_B_ receptor subunit mRNAs between 6 h and 6 months post-TBI in the hippocampus and thalamus. The expression of major GABA_A_ receptor subunit mRNAs (α1, α2, α5, β2, β3, γ2 and δ) was, often bilaterally, down-regulated in the GCL and in the CA3 pyramidal cells. Instead, expression of α4 (GCL, CA3, CA1), α5 (CA1) and γ2 (GCL, CA3, CA1) mRNA was up-regulated after 10 d and/or 4 months. Many of these changes were reversible. In the thalamus, we found decreases in α1, α4, β2, γ2 and δ mRNAs in the laterodorsal thalamus and in the area combining the posterior thalamic nuclear group, ventroposterolateral and ventroposteromedial complex at 6 h to 4 months post-TBI. Unlike in the hippocampus, thalamic subunit down-regulations were irreversible and limited to the ipsilateral side. However, contralaterally there was up-regulation of the subunits δ and α4 6 h and 4 months after TBI, respectively. PCR array analysis suggested a mild long-lasting GABA_A_ receptor channelopathy in the GCL and thalamus after TBI. Whereas TBI induces transient changes in the expression of GABA_A_ receptor subunits in the hippocampus (presumably representing compensatory mechanisms), alterations of GABA_A_ receptor subunit mRNAs in the thalamus are long-lasting and related to degeneration of receptor-containing neurons in thalamo-cortical relay nuclei.

This article is part of the Special Issue entitled ‘GABAergic Signaling in Health and Disease’.

## Highlights

• GABA_A_ receptor subunits are permanently lost in thalamic nuclei on the side of TBI.
• They are only transiently decreased in hippocampal subfields bilaterally.
• Subunit α4 is up-regulated in the thalamus and hippocampus contralateral to TBI.
• Efficacy of neurosteroids in preventing secondary epilepsy after TBI is suggested.

## 1 Introduction

Traumatic brain injury (TBI) is recognized as a major public health problem worldwide (Finfer and Cohen, 2001, Hyder et al., 2007). After initial brain damage caused by the direct mechanical force to the head, secondary neurodegeneration and other pathologies develop over the following days, weeks and months. These are accompanied by molecular changes and reorganization of neuronal networks (Loane and Faden, 2010, McAllister, 2011, Pitkanen and McIntosh, 2006). As a consequence of brain damage and of cellular plasticity, various co-morbidities, including post-traumatic epilepsy (PTE) can develop even years after the initial insult.

TBI has been estimated to cause about 20% of acquired epilepsy and 5% of all epilepsy (Herman, 2002). In patients with PTE, seizure onset is most often cortical, including frontal, parietal and polar/lateral temporal cortices (Gupta et al., 2014, Haltiner et al., 1997, Lowenstein, 2009) as well as the hippocampus (Mathern et al., 1994, Swartz et al., 2006). In addition to the damage at the site of cortical impact, patients with TBI can have remarkable degeneration in thalamic nuclei (Ross and Ebner, 1990, Ross et al., 1993, Thompson et al., 2005). For example, Ross et al. (1993) reported up to 75% reduction in the density of neurons in the reticular nucleus of the thalamus. Moreover, neuroimaging studies have revealed ongoing thalamic damage and microglial reactivity even 17 years after the initial TBI (Ramlackhansingh et al., 2011).

Similar to human TBI, histological and magnetic resonance imaging (MRI) studies revealed progressive hippocampal and thalamic damage over a one-year follow-up in rats with TBI induced by lateral fluid-percussion injury (FPI) (Hayward et al., 2010, Kharatishvili et al., 2007, Niskanen et al., 2013). Therefore, lateral FPI reproduces at least some aspects of the hippocampal and thalamic pathology observed in human TBI and consequently provides an opportunity to investigate the mechanisms and functional consequences of TBI in more detail (Kawai et al., 1995, Ross et al., 1995). Interestingly, lateral FPI was reported to induce marked changes in GABAergic transmission in the hippocampus. At 1 month post-TBI synaptic GABA_A_ receptor-mediated inhibition was profoundly reduced in the ipsilateral granule cells. Synaptic inhibition decreased over time, and by 6 months post-TBI became decreased also contralaterally (Pavlov et al., 2011). Progressive loss of synaptic inhibition paralleled with a decline in the number of parvalbumin (PV)-positive interneurons. On the other hand, GABA_A_ receptor subunit expression was largely unaltered at the chronic time points (Pavlov et al., 2011).

When investigating the hippocampal changes in the post-TBI expression of GABA_A_ receptor subunits, we observed marked abnormalities also in the expression of many GABA_A_ receptor subunits in thalamic nuclei. Moreover, we recently reported a marked reduction in PV/GABA containing neurons of the reticular nucleus of the thalamus (RT) after TBI (Huusko and Pitkanen, 2014). These studies kindled us to test further a hypothesis that progressive changes in GABA_A_ receptor subunits contribute to the development of post-TBI hyperexcitability. Consequently, the present study was designed to assess in more details the temporo-spatial evolution of abnormalities in 12 GABA_A_ receptor subunits over a 6 h to 4 months post-TBI follow-up. Moreover, we compared the receptor changes in the hippocampus and thalamus, the two areas showing pathologic changes after TBI and involved in candidate networks becoming epileptogenic after TBI.

## 2 Materials and methods

### 2.1 Animals

Adult male Sprague–Dawley rats (body weight 298–369 g at the time of injury; Harlan Netherlands B.V., Horst, Netherlands) were used in the study. The rats were individually housed in a controlled environment (temperature 22 ± 1 °C; 50–60% humidity; lights on from 07:00 to 19:00). They had access to food and water *ad libitum*. All animal experiments were conducted in accordance with the guidelines of the European Community Council Directives 86/609/EEC and approved by the Committee for Welfare of Laboratory Animals of the University of Eastern Finland.

**Supplementary Fig. 1:**
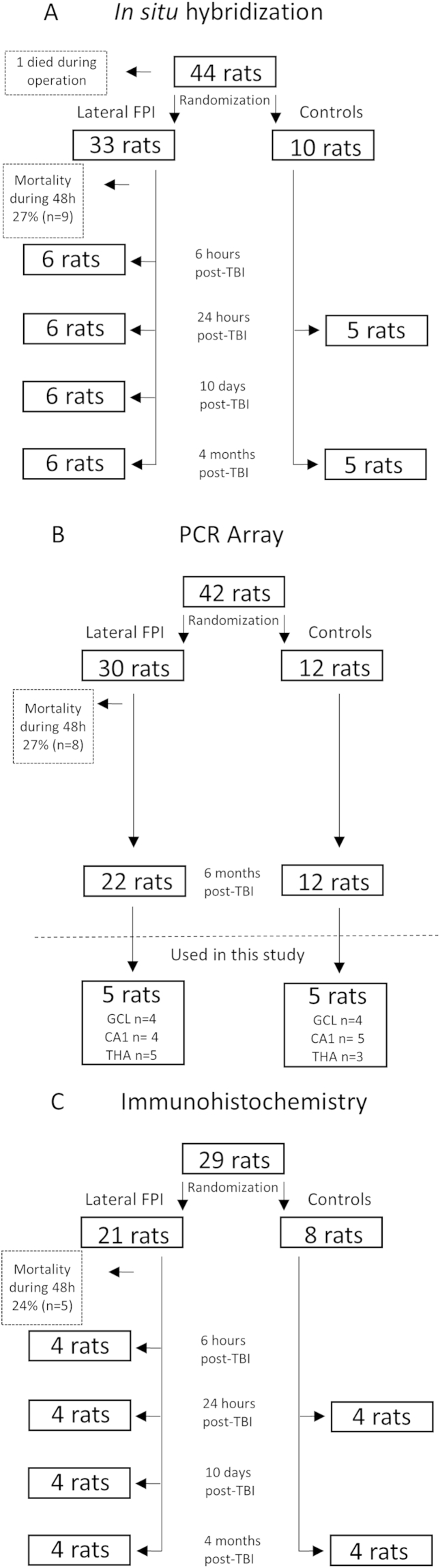
Flow-chart of rats included in (A) *in situ* hybridization, (B) PCR array study or (C) immunohistochemistry. Animals for PCR array analysis were randomly sampled from the bigger cohort. Abbreviations: GCL, granule cell layer; FPI, fluid-percussion injury; TBI, traumatic brain injury; THA, thalamus.

Traumatic brain injury (TBI) was induced using lateral FPI in three cohorts of animals from which 34 were anticipated for *in situ* hybridization, 30 for RT-PCR array and 21 for immunohistochemistry. From the surviving rats, in the present study 24 were used for *in situ* hybridization, 5 for RT-PCR array and 16 for immunohistochemistry (see Supplementary Fig. 1). Briefly, animals were anesthetized and placed in a Kopf stereotactic frame (David Kopf Instruments, Tujunga, CA, USA) and the skull was exposed. Thereafter, a circular craniectomy (Ø 5 mm) was performed over the left parietal lobe midway between lambda and bregma, leaving the dura intact. Lateral FPI was induced after connecting the rat to a fluid-percussion device (AmScien Instruments, Richmond, VA, USA). The mean severity of the impact was 3.45 ± 0.01 atm in a cohort used for *in situ* hybridization study, 3.38 ± 0.01 atm in a cohort used for PCR array study and 3.38 ± 0.02 atm in a cohort used for immunohistochemistry (no difference between the groups). Thirty rats underwent sham operation, that is, they underwent all surgical procedures without the exposure to impact, and were used as controls.

### 2.2 *In situ* hybridization

#### 2.2.1 Tissue processing

Rats were killed at 6 h, 24 h, 10 days, or 4 months (6 animals per group) after TBI by cervical dislocation. The brains were dissected and snap frozen by immersion in isopentane cooled to −70 °C. Isopentane was then allowed to evaporate at −70 °C, the brains were then sealed in plastic vials, and kept at −70 °C until further processed. Brains from control animals were sampled at 24 h (*n* = 5) and 4 months (*n* = 5) after sham-injury and processed accordingly. Consecutive coronar 20 μm sections were cut using a cryostat (Microm HM 560 M, Carl Zeiss AG, Vienna, Austria) at −20 °C, thaw-mounted on silane-coated slides, and stored at −70 °C. Every 11th section was stained with Cresyl violet, dehydrated, cleared in butyl acetate, and coverslipped using *Eukitt* mounting medium (O. Kindler GmbH, Freiburg, Germany). Cresyl violet stained sections were used to match the coronal levels in different rat brains to be sampled for *in situ* hybridization and histochemistry.

#### 2.2.2 *In situ* hybridization

Sections from each rat sampled from the same rostro-caudal level (AP −3.30 to −4.15 from the bregma for GABA_A_ receptor subunits, CCK, PV and GAD1 and AP −4.16 to −4.40 for GABA_B_ receptor subunits) were processed for *in situ* hybridization in the same incubation. The sequences of custom-synthesized oligonucleotides (Microsynth AG, Balgach, Switzerland) complementary to the respective mRNAs for GABA_A_ receptor subunits and two GABA_B_ receptor isoforms GABA_B1_ and GABA_B2_ have been listed previously (Drexel et al., 2013, Furtinger et al., 2003b, Tsunashima et al., 1997). As marker for GABA-ergic neurons of the reticular thalamic nucleus we determined glutamate decarboxylase1 (GAD1) and parvalbumin (PV) mRNAs and cholecystokinin-octapeptide (CCK) mRNA for principal neurons in the in the laterodorsal and posterior thalamic nuclei (for details see 2.2.3.). For PV mRNA the probe previously described was used (Drexel et al., 2011). For GAD1, an oligonucleotide complementary to bases 795–843 (NM_017007.1; CAC GGG TGC AAT TTC ATA TGT GAA CAT ATT GGT ATT GGC AGT TGA TGT C) and for CCK-8 an oligonucleotide complementary to bases 387–419 (NM_012829.2, GAA ATC CAT CCA GCC CAT GTA GTC CCG GTC ACT) of the respective rat genes was used.

*In situ* hybridization was performed as described previously (Tsunashima et al., 1997). Briefly, the oligonucleotides (2.5 pmol) were labeled at the 3ʹ-end with [^35^S] α-thio-dATP (1300 Ci/mmol; New England Nuclear, Boston, MA, USA) by reaction with terminal deoxynucleotidyltransferase (Roche Austria GmbH, Vienna, Austria) and precipitated with 75% ethanol and 0.4% NaCl. Frozen sections (20 μm) were immersed in ice-cold paraformaldehyde (2%) in phosphate-buffered saline (PBS), pH 7.2 for 10 min, rinsed in PBS, immersed in acetic anhydride (0.25% in 0.1 mol/l triethylamine hydrochloride) at room temperature for 10 min, dehydrated by ethanol series, and delipidated with chloroform. The sections were then hybridized in 50 μl hybridization buffer containing about 50 fmol (0.8–1 × 10^6^ cpm) labeled oligonucleotide probe for 18 h at 42 °C. The hybridization buffer consisted of 50% formamide (Merck, Darmstadt, Germany), 2xSSC (1xSSC consisting of 150 mmol/l NaCl and 15 mmol/l sodium citrate, pH 7.2). The sections were then washed twice in 50% formamide in 1xSSC (42 °C, 4 × 15 min), briefly rinsed in 1xSSC, rinsed in water, dipped in 70% ethanol, dried, and then exposed to BioMax MR films (Sigma–Aldrich, Vienna, Austria) together with [^14^C]-microscales (Amersham, Buckinghamshire, UK) for 14–28 d. Films were developed using Kodak D19 developer (Sigma–Aldrich, Vienna, Austria).

#### 2.2.3 Densitometric analysis of mRNA expression

Autoradiographic films were digitized and analyzed using NIH ImageJ software (version 1.46; U.S. National Institutes of Health, Bethesda, ML, USA; http://imagej.nih.gov/ij/). The 6 regions of interest (ROIs) included (i) granule cell layer of dentate gyrus, (ii) CA1 pyramidal cell layer of the hippocampus proper, (iii) CA3 pyramidal cell layer, (iv) laterodorsal (LD) thalamus, (v) area combining the posterior thalamic nuclear group (PO), ventroposterolateral (VPL) and ventroposteromedial (VPM) complex (PO/VPM/VPL), and (vi) reticular nucleus (RT) of the thalamus (Swanson, 1992).

Briefly, in the dentate gyrus or the hippocampus, a line (20 pixels wide) was drawn perpendicular to the layer of interest, and a density profile of gray values was plotted in ImageJ using the function “*Analyze* – *Plot profile*”. To assess gene expression in the thalamus, a circular region of interest (diameter 0.9–2.1 mm) was placed over the thalamic region of interest, and the mean gray values were plotted. Then, the relative optical densities (RODs) were calculated according to the following formula: ROD = log[256/(255 − mean gray value)]. Background RODs were calculated from gray values determined in the internal capsule of the same section and subtracted. Thereafter, it was confirmed that the range of RODs was within the linear range of autoradiography standards.

### 2.3 Expression of GABA_A_ receptor subunits, *Gad1* and *Gad2* by RT-PCR in the hippocampal principal cells and thalamus after TBI

#### 2.3.1 Tissue preparation

Rats (5 TBI and 5 controls) were deeply anesthetized with an intraperitoneal (i.p.) injection of a solution (6 ml/kg) containing sodium pentobarbital (58 mg/kg), chloral hydrate (60 mg/kg), magnesium sulfate (127.2 mg/kg), propylene glycol (42.8%) and absolute ethanol (11.6%) and transcardially perfused at 6 months after TBI with 0.9% NaCl (30 ml/min, 4 °C, for 4 min) to remove blood from the tissue. The brain was quickly removed from the skull, hemispheres were separated, and each one was placed to its own cryomold (#4557, Tissue-Tek, Sakura Finetek, Torrance, CA, USA) on ice (4 °C). Thereafter, ice cold optimum cutting temperature formulation (O.C.T., #4583, Tissue-Tek, Sakura Finetek) was added to the cryomolds to cover the entire sample. Cryomolds were snap frozen in isopentane, chilled with dry ice, and stored at −70 °C until further processed.

#### 2.3.2 Laser capture microdissection (LCM)

Coronal 8-μm-thick sections were cut with a cryostat (Leica CM3050 S, Leica Microsystems Nussloch GmbH, Germany) from the ipsilateral septal hippocampus at the level between −2.3 and −3.8 mm from the bregma (Swanson, 1992).

Twenty-five sections were randomly selected and moved to LCM slides (#11505151, Leica, MicroDissect GmbH, Herborn, Germany). Sections were stored at −70 °C for a maximum time of 48 h before further treatment.

Sections were stained with Cresyl violet (Merck, Darmstadt, Germany) to recognize different layers of the hippocampus. Briefly, sections were placed to 96% ethanol for 30 s, then 70% ethanol for 30 s, 50% ethanol for 30 s, and finally, to 1% Cresyl violet in 100% ethanol for 20 s. After that, sections were moved to 50% ethanol for 30 s, 70% ethanol for 30 s, 96% ethanol for 30 s and 100% ethanol for 30 s. Sections were rinsed in xylene twice for 5 min. Finally, slides were dried in a hood for 20 min.

LCM was performed immediately after Cresyl violet staining. The granule cell layer of the dentate gyrus and pyramidal layer of the CA1 were microdissected with a Leica LMD Laser Microdissection System (Leica, Wetzlar, Germany) in extraction buffer from the Arcturus^®^ PicoPure^®^ RNA Isolation Kit (Applied Biosystems, Foster City, CA, USA). Extraction buffer containing microdissected cells was vortexed and incubated for 30 min at 42 °C. After incubation, cell extract was centrifuged at 800 × *g* for 2 min and stored at −70 °C.

Methodology for microdissection of the thalamus has been described previously (Huusko and Pitkanen, 2014).

#### 2.3.3 RT-PCR array

RNA isolation was performed according to the manufacturer's protocol (PicoPure RNA User Guide, Part Number 12682-00ARC Rev A, Carlsbad, CA, USA). Endogenous DNA was removed using the RNase-Free DNase Set (Qiagen, Hilden, Germany). RNA concentration and quality was measured with a NanoDrop 1000 spectrophotometer (Thermo Fisher Scientific, Wilmington, DE, USA). First strand cDNA synthesis was done with an RT^2^ First Strand Kit (C-03, SABiosciences, Frederick, MD, USA) according to user manual (Part #1022A Version 5.01, SABiosciences) using 70 ng of RNA.

Gene expression of GABA_A_ receptor subunits, *Gad1* and *Gad2* was assessed using RT^2^Profiler™ PCR ArrayRat Neurotransmitter Receptors and Regulators array (PARN-060C, SABiosciences). Briefly, template cDNA was mixed with RT^2^SYBR Green/ROX qPCR Master Mix (SABiosciences) and moved to the RT^2^Profiler™ PCR Array plate (SABiosciences). The program for PCR was as follows (StepOne Software v2.1, Applied Biosystems, Foster City, CA, USA): 1 cycle (95 °C, 10 min), 40 cycles (95 °C, 15 s; 60 °C, 60 s) in a StepOnePlus™ Real-Time PCR System (Applied Biosystems).

### 2.4 Immunohistochemistry

#### 2.4.1 Tissue processing

Rats were perfused for immunohistochemistry at 6 h, 24 h, 10 d, or 4 months after TBI (four rats per group). Control animals were perfused at 24 h or 4 months (4 rats each time point) after sham-operation. Briefly, rats were deeply anesthetized with an i.p. injection of the same solution used for animals in a cohort of rats for PCR array and perfused through the ascending aorta with 50 ml PBS (50 mM phosphate buffer, pH 7.4, in 0.9% NaCl) followed by 200 ml 4% paraformaldehyde in PBS as described previously (Drexel et al., 2011). Brains were immediately removed from the skull and postfixed in the same fixative for 90 min at 4–8 °C. Subsequently brains were cryoprotected in 20% sucrose in 0.02 M PBS (pH 7.4) at 4–8 °C for 36 h. Brains were sealed in vials containing 20% sucrose and sent from Kuopio to Innsbruck. There the brains were rapidly frozen by immersion in −70 °C isopentane for 3 min. After letting the isopentane evaporate, brains were stored in tightly sealed vials at −70 °C.

#### 2.4.2 Immunohistochemistry

Immunohistochemistry for GABA_A_ receptor subunits was done for free floating sections (30-μm-thick) as described previously (Pirker et al., 2000, Sperk et al., 1997). Affinity purified antisera raised in rabbits against fusion proteins of peptide sequences (specific for the individual subunits) were used at the given concentrations and as described in detail previously (Pirker et al., 2000, Sperk et al., 1997). Goat anti-rabbit antibody coupled to horse radish peroxidase (P0448, Daco, Vienna) was used as secondary antibody (1:250) with subsequent labeling by reaction with 3,3-diaminobenzidine.

### 2.5 Statistical analysis

*In situ* hybridization data was analyzed using GraphPad Prism 5.0a for Macintosh (GraphPad Software, San Diego, CA, USA). Analysis of variance (ANOVA) with Dunnett's multiple comparison *post hoc* test was used to analyze between-group differences among multiple sets of data. All data are presented as mean ± SEM. PCR Array analysis was done using a web-based data analysis tool (http://www.sabiosciences.com/pcrarraydataanalysis.php). Unsupervised hierarchical clustering of the GABA_A_ receptor subunits, *Gad1* and *Gad2* was performed using the same data analysis tool and is presented as a heat map with dendrograms indicating co-regulated genes across groups or individual samples. Statistical significance was defined as *p* < 0.05.

## 3 Results

### 3.1 Mortality

Mortality during first 48 h after TBI was 26% (9/33) in a cohort of animals for *in situ* hybridization, 27% (8/30) in a cohort of the animals used for PCR array study and 25% (5/20) in a cohort of animals used for immunohistochemistry (see Supplementary Fig. 1).

### 3.2 Neurodegeneration and regulation of expression of GABA_A_ receptor subunits in the hippocampus after FPI

#### 3.2.1 Neurodegeneration

**Fig. 1:**
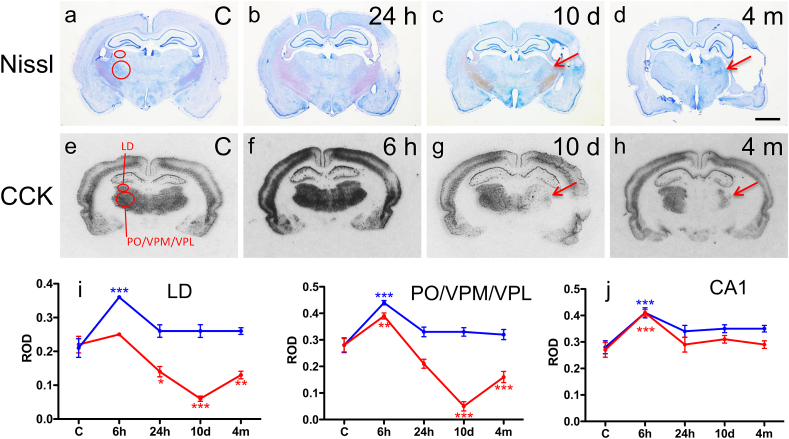
Severe neuronal damage in the thalamus but not in the hippocampus after lateral FPI. (a–d) Nissl staining revealed shrinkage of the hippocampus which was associated with a minor loss of principal cells. The thalamus, however, showed a clear damage already at 24 h post-TBI which progressed over the 4-month follow-up. (e–j) Quantitative assessment of CCK mRNA. No change was observed in the hippocampus. However, we found a decrease in the ipsilateral thalamus already at 24 h post-TBI (red; i), progressing over the 4-months follow-up. In the contralateral thalamus (blue; i), we found increased level of CCK mRNA at 6 h post-TBI as compared to controls which normalized by 24 h post-TBI. In the CA1 pyramidal cell layer of the hippocampus there was no decrease in CCK mRNA (e–h, j). The LD and PO/VPM/VPL groups are outlined in control sections (a, e). Arrows denote damage in the thalamic nuclei. Scale bar = 250 μm (a–h). Statistical significance: *p < 0.05, **p < 0.01, ***p < 0.001 as compared to controls. Abbreviations: CCK, cholecystokinin; LD, laterodorsal thalamic nucleus; PO, posterior thalamic nuclear group; VPM, ventroposteromedial nucleus; VPL, ventroposterolateral nucleus. (For interpretation of the references to color in this figure legend, the reader is referred to the web version of this article.)

Analysis of Nissl-stained preparations revealed hippocampal shrinkage ipsilaterally at 10 d and 4 months post-TBI (Fig. 1c–d). However, this was not associated with any remarkable loss of principal cells in the dentate gyrus or in the hippocampus proper. Accordingly, semiquantitative analysis of expression of CCK mRNA as a marker of CA1 pyramidal cells (Gruber et al., 1993) showed that the level of expression at 10 d and 4 months was comparable between injured and control rats both ipsilaterally and contralaterally (Fig. 1j). Interestingly, however, we found a temporary up-regulation of CCK mRNA in the CA1 at 6 h post-TBI both ipsilaterally (147 ± 4% as compared to that in controls, *p* < 0.001; Fig. 1f) and contralaterally (136 ± 6%, *p* < 0.001; Fig. 1f).

#### 3.2.2 Expression of GABA_A_ receptor subunits

**Fig. 2:**
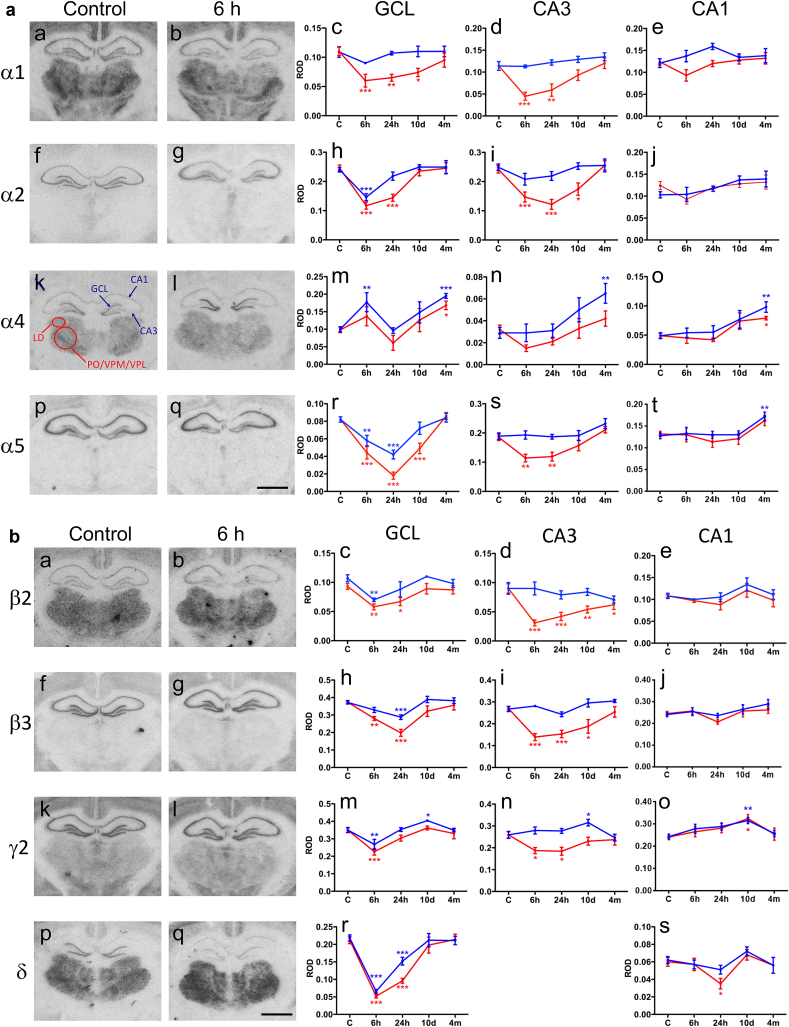
a and b. Time courses of changes in the expression of GABA_A_ receptor subunit mRNAs in the hippocampus and thalamus (α1, α2, α4, α5, β2, β3, γ2 and δ). (2a: a–b; f–g; k–l, p–q and 2b: a–b; f–g; k–l, p–q) Representative photomicrographs demonstrating the level of GABA_A_ receptor subunit mRNAs in the hippocampus. (2a: c–e; h–j; m–o, r–t and 2b: c–e; h–j; m–o, r–s) Optic densities for mRNA levels were determined by image analysis (see Methods) of film autoradiographs after *in situ* hybridization. Relative optic densities (ROD) in the granule cell layer (GCL) and pyramidal cell layers are depicted as red lines for ipsilateral side after TBI and as blue lines for contralateral side after TBI. In k the areas of interest examined are indicated in a representative way for the laterodorsal nucleus (LD) and the area combining the posterior thalamic nuclear group (PO), ventroposterolateral (VPL) and ventroposteromedial (VPM) complex (PO/VPM/VPL) (Swanson, 1992) with red circles and the location where the line scans were set are shown with blue arrows for GCL and hippocampal subfields CA1 and CA3 (nomenclature according to Swanson, 1992). Note that mRNA levels for all other subunits except α4 decrease bilaterally at the early time intervals after TBI (6 h and/or 24 h). However, the mRNA levels mainly recover close to control values on both sides within 4 months. Subunit γ2 mRNA level increases 10 days and levels of α4 and α5 4 months after TBI in sector CA1 indicating reflectory adaptation. Scale bar = 250 μm (a–b, f–g. k–l, p–q). Statistical significance: *p < 0.05, **p < 0.01, ***p < 0.001 as compared to controls. Abbreviations: GCL, granule cell layer. (For interpretation of the references to color in this figure legend, the reader is referred to the web version of this article.)

Time course of changes in the expression of GABA_A_ receptor subunit mRNAs in the different hippocampal subfields is summarized in Fig. 2a and b. The most pronounced changes were seen in the granule cell layer of the ipsilateral dentate gyrus, in which the TBI induced a prominent decrease in the expression of mRNAs for α1 (55 ± 10% of that in controls, *p* < 0.001), α2 (48 ± 4%, *p* < 0.001), α5 (21 ± 5%, *p* < 0.001), β2 (62 ± 5%, *p* < 0.01), β3 (53 ± 5%, *p* < 0.001), and δ (25 ± 2% *p* < 0.001) subunits already at 6 and 24 h post-TBI. Expression of γ2 subunit was decreased at 6 h (65 ± 5%, *p* < 0.001) post-TBI. Importantly, the expression of all GABA_A_ receptor subunit mRNAs returned to control level by 4 months post-TBI. Interestingly, comparable transient down-regulation (30–77% of that in controls, *p* < 0.01) of GABA_A_ receptor subunits (except α1 mRNA) was also found contralaterally in the granule cell layer. Unlike the other GABA_A_ receptor subunits, the level of α4 mRNA was increased at 6 h (ipsilaterally: 140 ± 28% as compared to that in controls, *p* > 0.05; contralaterally: 177 ± 27% as compared to controls, *p* < 0.01) and 4 months (ipsilaterally: 171 ± 12%, *p* < 0.05; contralaterally: 195 ± 7%, *p* < 0.001) post-TBI in granule cell layer both ipsilaterally and contralaterally.

Similar to granule cell layer, we found a transient down-regulation in the expression of α1 (40 ± 8% of that in controls, *p* < 0.001), α2 (50 ± 8%, *p* < 0.001), α5 (62  ± 7%, *p* < 0.01), β2 (34 ± 6%, *p* < 0.001), β3 (52 ± 6%, *p* < 0.001) and γ2 (71 ± 7%, *p* < 0.05) mRNAs in the CA3 pyramidal cell layer at 6 h and 10 d post-injury (Fig. 2a and b). Contralaterally, the subunit expression was comparable to that in controls except of that of α4. It showed a tendency to be bilaterally up-regulated in sectors CA3 and CA1 at 10 d (*p* > 0.05) and was up-regulated bilaterally (CA3 ipsilaterally: 131 ± 22% of that in controls, not significant; CA3 contralaterally: 224 ± 31%, *p* < 0.01; CA1 ipsilaterally: 161 ± 6%, *p* < 0.05; CA1 contralaterally: 200 ± 18%, *p* < 0.01) at 4 months post-TBI. Also the expression of α5 mRNA was up-regulated contralaterally in sector CA1 at 4 months post-TBI (134 ± 8% of that in controls, *p* < 0.01) (Fig. 2a).

### 3.3 Neurodegeneration and regulation of expression of GABA_A_ receptor subunits in the thalamus

#### 3.3.1 Neurodegeneration

Analysis of Nissl-stained sections revealed progressive damage in the LD and PO/VPM/VPL starting at 24 h post-TBI (Fig. 1b–d). Accordingly, expression of CCK mRNA at 24 h post-injury was reduced to 64 ± 7% of that in controls in the LD (*p* < 0.05) and there was a trend towards reduced levels also in the PO/VPM/VPL (75 ± 6% of that in controls, *p* > 0.05). The loss progressed to almost entire reduction (to 59 ± 5% of that in controls in the LD after 4 months (*p* < 0.01) and to 18 ± 6% in the PO/VPM/VPL after 10 d, *p* < 0.001) of the thalamic nuclei ipsilateral to TBI after 10 d and 4 months (Fig. 1g–h). Contralateral to TBI, levels of CCK mRNA were comparable to that in controls both in the LD and PO/VPM/VPL at the late time intervals. However, similar to the hippocampus, we found a remarkable up-regulation of CCK mRNA at 6 h post-TBI in the PO/VPM/VPL ipsilaterally (139 ± 4% as compared to that in controls, *p* < 0.001), and both in the LD (171 ± 2%, *p* < 0.001) and PO/VPM/VPL contralaterally (157 ± 3%, *p* < 0.001; Fig. 1i).

#### 3.3.2 Expression of GABA_A_ receptor subunits

GABA_A_ receptor subunits were differently distributed in the thalamic nuclei investigated. Whereas the LD and PO/VPM/VPL complex showed a high level of expression of α1, α4, β2, γ2 and δ subunits, the reticular nucleus was rich of α3, β3 and γ2 subunits (Sperk et al., 1997, Tsunashima et al., 1997).

**Fig. 3:**
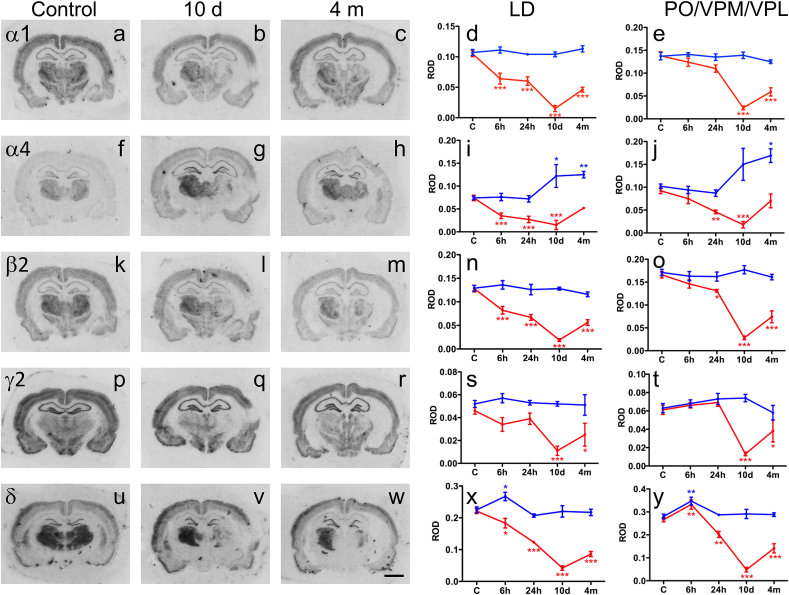
Time courses of changes in the expression of GABA_A_ receptor subunit mRNAs that are primarily expressed in the LD and PO/VPM/VPL complex (α1, α4, β2, γ2 and δ). (a–c, f–h, k–m, p–r, u–w) On the left side we present representative images of film autoradiographs after *in situ* hybridization for the respective GABA_A_ receptor subunit mRNAs. (d–e, i–j, n–o, s–t, x–y) Quantitative evaluation of relative optic densities (ROD) and respective statistics are shown on the right panels. ROD values are depicted as red (ipsilateral to TBI) and blue (contralateral to TBI) lines. Note the rapid decreases in most subunit mRNAs ipsilaterally. Interestingly, the mRNA expression remains mostly unchanged in the contralateral side. Significant increases were observed for the α4 subunit mRNA on the contralateral side at the late intervals and for δ subunit mRNA in both hemispheres after 6 h, respectively. Scale bar = 250 μm (a–c, f–h, k–m, p–r, u–w). Statistical significance: *p < 0.05, **p < 0.01, ***p < 0.001 as compared to controls. Abbreviations: LD, laterodorsal thalamic nucleus; PO, posterior thalamic nuclear group; VPM, ventroposteromedial nucleus; VPL, ventroposterolateral nucleus. (For interpretation of the references to color in this figure legend, the reader is referred to the web version of this article.)

Representative examples of film autoradiographs showing the distribution of GABA_A_ receptor subunit mRNAs in the LD and PO/VPM/VPL at 10 d and 4 months post-TBI are shown in Fig. 3. On the injured side there was a progressive decrease of 5 GABA_A_ receptor subunit mRNAs for up to 10 days post-TBI (Fig. 3). Interestingly the decrease in mRNAs of different subunits was not uniform. At 6 h and 24 h post-TBI there were no remarkable decreases in α1, β2 or γ2 mRNAs in the PO/VPM/VPL (Fig. 3e, o, t) and in γ2 mRNA in the LD (Fig. 3s). On the other hand, levels of α1, α4 and β2 decreased to 35–55% of that in controls in the LD already at 6 h–24 h post-TBI (*p* < 0.001)(Fig. 3d, i, n). At 4 months post-TBI, however, the levels of all subunit mRNAs were increased towards the control levels. The levels of α4 mRNA had reached the control level in the both nuclei (Fig. 3i, j). Contralaterally, the levels of α1, β2 and γ2 did not change over the 4-months follow-up. However, at 10 d and 4 months post-TBI we found an increase in the expression of α4 mRNA in the LD (165 ± 34%, *p* < 0.05 and 169 ± 9%, *p* < 0.01) as well as in the PO/VPM/VPL (147 ± 34%, *p* > 0.05, and 166 ± 15%, *p* < 0.05) (Fig. 2i, j). Similarly, δ mRNA was transiently up-regulated 6 h (LD: 119 ± 6%, *p* < 0.05; PO/VPM/VPL: 124 ± 6% as compared to controls, *p* < 0.01) after TBI.

**Fig. 4:**
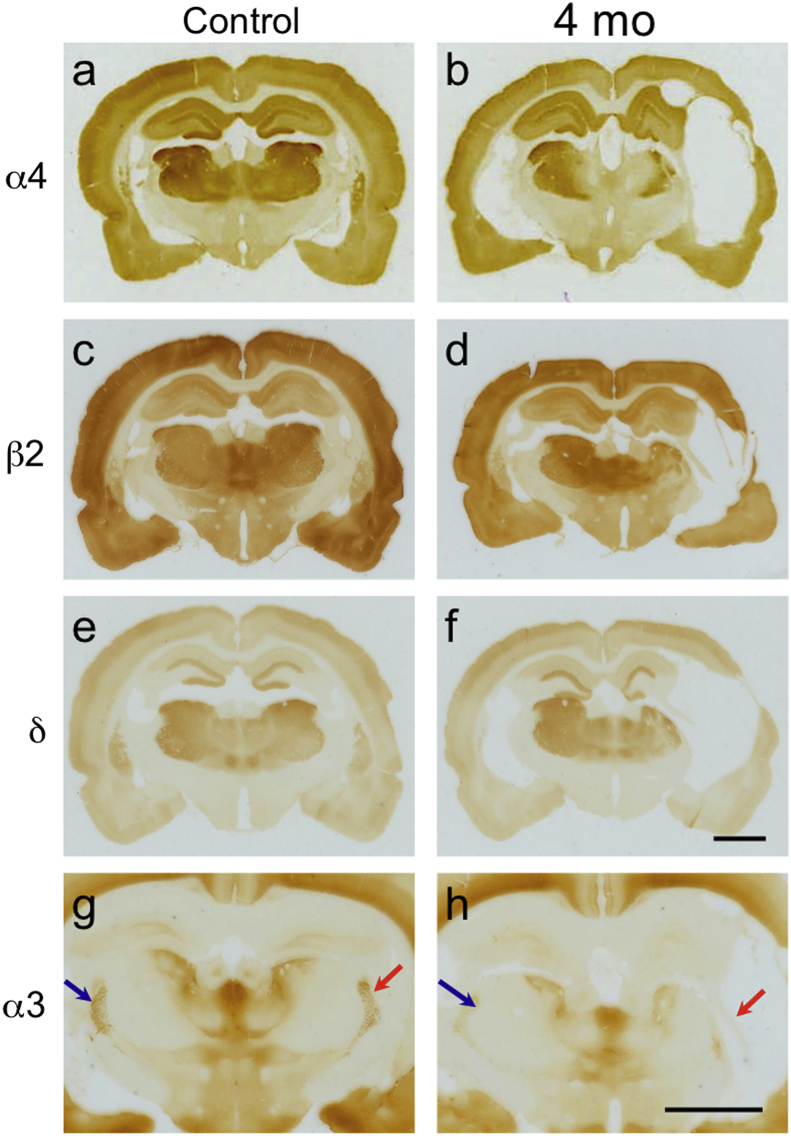
Changes in the immunoreactivities for subunits α4, β2, δ and α3 after lateral FPI. (a, c, e, g) Representative immunostained sections from a control rat (b, d, f) Note the persistent decreases in α4, β2 and δ subunit expression 4 months after TBI in the thalamic areas investigated. There was also a clear increase in α4 subunit protein in the LD and PO/VPM/VPL on the side contralateral to the injury (b). Arrows in g and h denote labeling of the reticular thalamic nucleus for α3 immunoreactivity. Scale bars in f (for a–f) and in h (for g and h): 250 μm.

Fig. 4 shows examples of immunostainings for α4, β2 and δ subunits. In line with *in situ* hybridization, we found a persistent decrease in labeling of α4, β2 and δ subunit expression at 4 months post-TBI in the LD and PO/VPM/VPL (Fig. 4b, d, f). As expected, immunoreactivity for α4 subunit was increased in the contralateral LD and PO/VPM/VPL (Fig. 4b).

### 3.4 Expression of GABA_B_ receptor subunits is affected in the thalamus but not in the hippocampus

**Fig. 5:**
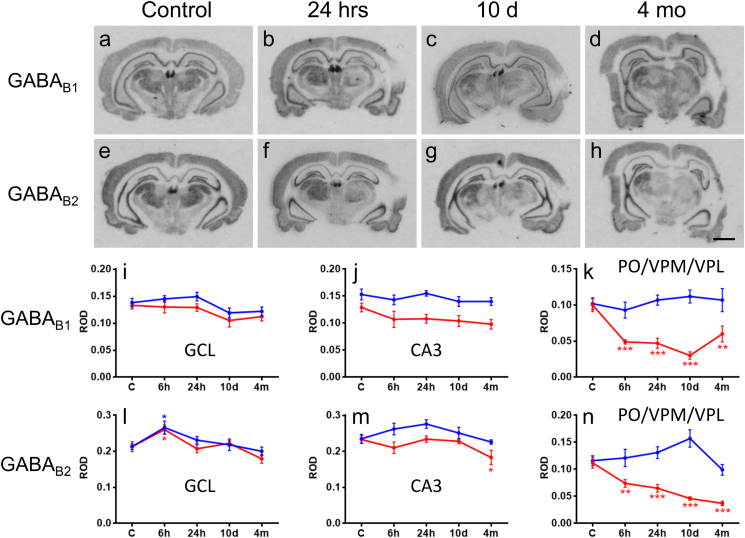
Time courses of changes in the expression of GABA_B1_ (GBBR1) and GABA_B2_ (GBBR2) receptor subunit mRNAs in the hippocampus and LD and PO/VPM/VPL nuclei of the thalamus after TBI. Panels a–d and e–h show photomicrographs of autoradiographies after *in situ* hybridization for GABA_B1_ and GABA_B2_, respectively. Analysis was done from sections caudal to those used for analysis of GABA_A_ receptor subunits and GAD1. Panels I–n show the respective relative optic densities (ROD) in the granule cell layer (GC) and CA1 pyramidal cell layer of the hippocampus and in the PO/VPM/VPL. Besides a transient bilateral up-regulation of GABA_B2_ mRNA in the granule cell layer 6 h after TBI, we did not observe a significant change in GABA_B_ receptor subunits in the hippocampus during the 4-months follow-up after TBI. The marked unilateral decrease of GABA_B1_ and GABA_B2_ subunit mRNAs in the LD and PO/VPM/VPL likely reflects underlying neuronal damage in these areas. Scale bar in h: 250 μm. Statistics: *p < 0.05, **p < 0.01, ***p < 0.001 vs. control.

We also investigated mRNA levels of the two GABA_B_ receptor isoforms, GABA_B1_ and GABA_B2_ in the thalamus and the hippocampus. Both receptors are thought to be present as heterodimers in the membrane (Gassmann and Bettler, 2012). Analysis was done in sections caudal to those used for analysis of GABA_A_ receptor subunits, and contained either PO alone or the entire PO/VPM/VPL complex. Expression of subunit mRNAs did not differ between PO and VPM/VPL. Consequently, the three nuclei were analyzed together as we pooled the data of our measurements. In the ipsilateral granule cell layer, we found a transient increase (122 ± 6% of that in controls, *p* < 0.05) in GABA_B2_ receptor mRNA at 6 h and in the CA3 a mild decrease (79 ± 9% as compared to controls, *p* < 0.05) at 4 months post-TBI (Fig. 5). Contralaterally, only a transient increase of the GABA_B2_ mRNA was found at 6 h post-TBI (125 ± 8% of that in controls, *p* < 0.05; Fig. 5). There was decreased expression of GABA_B1_ and GABA_B2_ receptors in the PO/VPM/VPL (30–66% of that in controls) ipsilaterally in all investigated time points after TBI (*p* < 0.01).

### 3.5 Expression of GAD1, PV and of subunit α3 mRNAs in the reticular nucleus of the thalamus

**Fig. 6:**
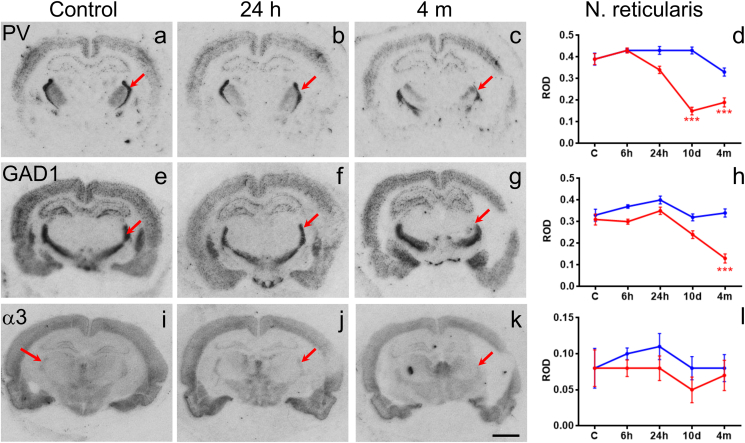
Changes in the expression of parvalbumin, glutamate decarboxylase1 (GAD1) and the α3 subunit mRNAs in the reticular nucleus of the thalamus. Panels on the left show autoradiographic images of *in situ* hybridization for parvalbumin, glutamate decarboxylase1 (GAD1) and the α3 subunit mRNAs. Right side panels show relative optic densities (ROD) in the reticular thalamic nucleus (arrows). Note the decreases in all three mRNAs ipsilateral to TBI. Scale bar = 250 μm (a–c, e–g, i–k). Statistical significance: *p < 0.05, ***p < 0.001 as compared to controls. Abbreviations: GAD1, Glutamate decarboxylase1; PV, parvalbumin; RT, reticular nucleus of the thalamus.

To expand our previous studies in the hippocampus and thalamus (Huusko and Pitkanen, 2014, Pavlov et al., 2011), we investigated two presynaptic (PV and GAD1 mRNAs) and one postsynaptic marker (α3 mRNA) of GABAergic circuitries in the RT. Like in the hippocampus, the mRNA levels of PV and GAD1 were only 40–50% (10 d and 4 m) and about 40% (4 m) of that in controls in the RT, respectively (*p* < 0.001; Fig. 6d, h). In contrast, there was no significant change in α3 mRNA expression (Fig. 6l).

### 3.6 RT-PCR array analysis of GABA_A_ receptor subunits, *Gad1* and *Gad2* at 6 months post-TBI

In the granule cell layer, we found a 1.2-fold up-regulation of the expression of *Gabrb3* (*p* < 0.05; Table 1). In addition, both *Gad1* and *Gad2* genes were down-regulated (0.66-fold, *p* < 0.05 and 0.60, *p* < 0.01; respectively). In the pyramidal cell layer of the CA1, we did not find any changes in the expression of GABA_A_ receptor subunits, *Gad1* or *Gad2* (Table 1).

**Table 1:** Expression of GABA_A_ receptor subunits, *Gad1* and *Gad2* at 6 months after TBI.

Subfield	Gene code	Fold change	*p*-value
Granule cell layer	Gabra1	1.12	0.348
	Gabra2	1.11	0.439
	Gabra3	1.13	0.417
	Gabra4	1.00	0.931
	Gabra5	1.05	0.674
	Gabra6	0.80	0.690
	Gabrb2	1.04	0.617
	Gabrb3	1.17*	0.036
	Gabrd	1.32	0.058
	Gabre	2.78	0.105
	Gabrg1	1.95	0.107
	Gabrg2	1.13	0.127
	Gabrp	0.74	0.788
	Gabrq	3.78	0.327
	Gabrr1	0.96	0.639
	Gabrr2	0.91	0.848
	Gad1	0.66*	0.024
	Gad2	0.60**	0.001
Pyramidal cell layer of the CA1	Gabra1	0.71	0.201
	Gabra2	0.91	0.571
	Gabra3	0.81	0.355
	Gabra4	0.73	0.239
	Gabra5	0.73	0.115
	Gabra6	0.82	0.438
	Gabrb2	0.74	0.461
	Gabrb3	0.72	0.204
	Gabrd	0.74	0.356
	Gabre	0.60	0.405
	Gabrg1	0.92	0.560
	Gabrg2	0.78	0.234
	Gabrp	0.36	0.238
	Gabrq	0.50	0.325
	Gabrr1	0.63	0.150
	Gabrr2	0.77	0.814
	Gad1	0.89	0.509
	Gad2	0.85	0.421
Thalamus	Gad1	0.70*	0.013
	Gad2	0.84	0.154

Statistical significance: *p < 0.05; **p < 0.01, as compared to controls.

Results from the gene expression analysis of the GABA_A_ receptor subunits in the thalamus (down-regulation of *ε* and *θ*) have been presented elsewhere (Huusko and Pitkanen, 2014). Reanalysis of data on array revealed also a 0.70-fold down-regulation of *Gad1* (*p* < 0.05) in the thalamus (Table 1).

**Fig. 7:**
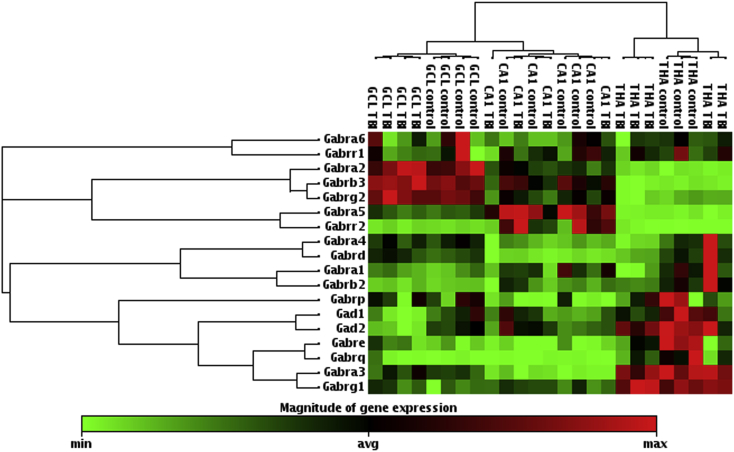
Unsupervised hierarchical clustering of gene expression levels of 16 GABA_A_ receptor subunits, *Gad1* and *Gad2* in controls and TBI animals at 6 months after insult when using join type minimum. A heat map presents a level of gene expression of individual genes in the different brain areas. Every measured brain area had a unique gene expression pattern of GABA_A_ receptor subunits. A dendrogram in the left side of the figure indicates clustered subunits. Similarly, a dendrogram in the upper part of the figure shows clustering results between individual animals, groups and the brain areas. Note that in the granule cell layer, the analysis separated TBI animals from controls. In addition, in the thalamus, we found also a trend for separation. We did not find any difference in the CA1 between TBI animals and controls. Abbreviations: GCL, granule cell layer; *Gabra1*, Gamma-aminobutyric acid receptor subunit alpha-1; *Gabra2*, Gamma-aminobutyric acid receptor subunit alpha-2; *Gabra3*, Gamma-aminobutyric acid receptor subunit alpha-3; *Gabra4*, Gamma-aminobutyric acid receptor subunit alpha-4; *Gabra5*, Gamma-aminobutyric acid receptor subunit alpha-5; *Gabra6*, Gamma-aminobutyric acid receptor subunit alpha-6; *Gabrb2*, Gamma-aminobutyric acid receptor subunit beta-2; *Gabrb3*, Gamma-aminobutyric acid receptor subunit beta-3; *Gabrd*; Gamma-aminobutyric acid receptor subunit delta; *Gabre*, Gamma-aminobutyric acid receptor subunit epsilon; *Gabrg1*, Gamma-aminobutyric acid receptor subunit gamma-1; *Gabrg2*, Gamma-aminobutyric acid receptor subunit gamma-2; *Gabrp*, Gamma-aminobutyric acid receptor subunit pi; *Gabrr1*, Gamma-aminobutyric acid receptor subunit rho-1; *Gabrr2*, Gamma-aminobutyric acid receptor subunit rho-2; *Gabrq*, Gamma-aminobutyric acid receptor subunit theta; *Gad1*, Glutamate decarboxylase1 (GAD67); *Gad2*, Glutamate decarboxylase 2 (GAD65); THA, thalamus.

Interestingly, when individual subunits were analyzed separately, only few of them reached the level of statistical significance after TBI as compared to controls. However, when we performed unsupervised hierarchical clustering analysis of all GABA_A_ receptor subunits, *Gad1* and *Gad2*, we were able to see a pattern in the change of subunit gene expression level after TBI, particularly in the granule cell layer (Fig. 7). Noteworthy, clustering analysis also separated granule cell layer, CA1 and thalamus according the GABA_A_ receptor subunit gene expression level.

## 4 Discussion

The loss of presynaptic GABAergic terminals has been shown to result in reduced neurotransmitter release and abnormal expression of GABA_A_ receptor subunits in postsynaptic neurons, which can contribute to the neuronal hyperexcitability (Zhang et al., 2007). Moreover, analysis of the tissue from patients with temporal lobe epilepsy (TLE) has revealed interneuron loss which is associated with changes in the expression of GABA_A_ receptor subunits (Loup et al., 2000, Pirker et al., 2003). Based on these data, we hypothesized those progressive changes in GABA receptor subunits can contribute to the development of post-TBI hyperexcitability. We have five main findings: 1) There was a transient bilateral down-regulation of different GABA_A_ receptor subunits in the dentate gyrus and hippocampal subfields. Only exception was α4-subunit which gene expression was up-regulated in acute and chronic time points after TBI. 2) In the thalamus the down-regulation of the subunit genes progressed ipsilaterally towards longer time points after insult. 3) There was a down-regulation of GABA_B1_ and GABA_B2_ in the thalamus after TBI. The mRNA levels assessed from granule cell layer or CA3 subfield of the hippocampus were close to control values in all investigated time points. 4) As consistent to our previous study, the levels of the PV and GAD1 mRNA were lower in the RT as compared to controls. 5) PCR array analysis of the GABA_A_ receptor subunits reproduced well the findings obtained using the *in situ* hybridization. Only few significant changes in the subunit level were found. However, interestingly, the clustering analysis revealed different patterns in the subunit gene expression between TBI animals and controls in the granule cell layer of the dentate gyrus and thalamus suggesting mild, long-lasting channelopathy after TBI.

### 4.1 Alterations in GABA_A_ receptor subunit gene expression is long-lasting in the thalamus but transient in the hippocampus after lateral FPI

The GABA_A_ receptors are the main mediator of fast inhibitory neurotransmission in the adult central nervous system (CNS) (Mohler et al., 1998). The receptor is a 275 kDA heteropentameric glycoprotein formed by five different subunits (α 1–6, β 1–3, γ 1–3, δ, ρ 1–3, ε, θ, π) which properties depend on the subunit combination (Campagna-Slater and Weaver, 2007, Fritschy and Brunig, 2003, Mohler et al., 1998, Sieghart and Sperk, 2002). TBI caused rapid (6–24 h (after 6–24 h) decreases in all major subunits α1, α2, α5, β1, β2, β3, γ2 and δ (with the exception of α4) in the dentate gyrus and in sector CA3 of the hippocampus and in the thalamus (subunits α1, α4, β2, and δ with the exception of γ2 in LD and PO/VPM/VPL and β2 in PO/VPM/VPL) at the injured side. Whereas in the dentate gyrus and hippocampus proper the expression of all subunits investigated recovered already after 10 days back to control level decreases in GABA_A_ receptor subunits progressed and were maximal (up to 85%) 10 days after lateral FPI.

Analysis of tissue resections from patients with TLE have revealed altered GABA_A_ receptor subunit expression patterns i.e. there is greater labeling of the α2, γ2, β2 and β3 subunits (Loup et al., 2000, Pirker et al., 2003). Many laboratories have demonstrated the altered expression of GABA_A_ receptor subunits in rat models of acquired epilepsy induced by SE (Brooks-Kayal et al., 1998, Friedman et al., 1994, Fritschy et al., 1999, Schwarzer et al., 1997, Sperk et al., 1998, Tsunashima et al., 1997). However, the gene expression patterns have been found to depend on the SE model used and the time point post-SE when sampling was done. In addition to our previous studies (Huusko and Pitkanen, 2014, Pavlov et al., 2011), three other laboratories have investigated acquired GABA_A_ receptor channelopathy after TBI. Gibson et al. (2010) induced TBI by using central FPI in adult Sprague–Dawley rats. These investigators measured the levels of the hippocampal GABA_A_ receptors using western blot at 3 h, 6 h, 24 h or 7 d post-TBI. The level of α1 was increased by about 125% at 3 h and 6 h post-TBI of that in controls, but later it declined (50%). The protein level of the α3 was reduced to 75% only at 24 h post-TBI. The β3 level was first (3 h post-TBI) decreased to about 50% of that in controls but later (6–24 h) it became increased (130%). Finally, at 7 d post-TBI the level of the β3 became comparable to that in controls. In addition, the protein level of the γ2 was considerably increased (200% of that in controls) at 3 h post-TBI, resembling the control values more closely at later time points, except at 24 h post-TBI when it was only about 30% of that in controls. These changes were inhibited by injections of the NMDA receptor antagonist MK-801 prior to TBI. Raible et al. (2012) induced TBI using lateral FPI in adult Sprague–Dawley rats. They found reduced levels of the α1-subunit mRNA in whole hippocampal homogenates at 6 h post-TBI and reduced levels of α1-subunit protein at 24 h, 48 h and 1 wk post-TBI. Interestingly, the levels of the α4-subunit mRNA were increased at 6 h post-TBI but an increase in the protein level was found only at 24 h post-TBI, as the levels were below those of controls at 1 wk post-TBI. The study of Kharlamov et al. (2011) compared the hippocampal protein values of the α1, α4, *γ2* and δ subunits between controls, post-CCI Sprague–Dawley rats with or without spontaneous seizures at 7–11 months post-TBI. These investigators found a decreased protein expression level of γ2 in the rat group with PTE but an increased level of δ in the subgroup of rats without seizures. Thus the increased α4-subunit could be assembled with a δ-subunit. Also α4-containing GABA_A_ receptors without a γ2 or δ subunit may be possible (Bencsits et al., 1999). How thalamic subunit changes presented here are comparable to other TBI models remains to be studied as there is no literature available.

We and other laboratories have shown a robust loss of presynaptic interneurons in both the hippocampus and thalamus after experimental TBI (Huusko and Pitkanen, 2014, Huusko et al., 2013, Pavlov et al., 2011, Toth et al., 1997, Zhang et al., 2011a, Zhang et al., 2011b). Interestingly, however, surviving principal neurons of the hippocampus still maintain the normal gene expression of the GABA_A_ receptor subunits although GABAergic transmission is impaired mainly due to presynaptic mechanisms (Pavlov et al., 2011). In the thalamus, a massive neuronal loss (this study, Huusko and Pitkanen, 2014) can be a major cause of decline in GABA_A_ receptor subunit mRNA levels ipsilaterally. However, to some extent a down-regulation of the subunit gene expression may contribute to the decreasing mRNA levels. Further, early changes in GABA_A_ receptor subunit composition can indicate an impairment of GABAergic transmission in the post-TBI tissue and may contribute to its progressing neurodegeneration i.e. the extensive loss of PV and GAD1 positive neurons in the RT may contribute to a pre- and post-synaptic run-down of inhibition. Both, GAD1 and PV mRNA levels were reduced in the RT after TBI consistent with the severe damage seen also in the RT. It substantiates the severe unilateral loss of GABA-ergic input to other thalamic nuclei in TBI, which may be causatively related to the damage in these areas.

The γ2-subunit, together with gephyrin and GABA_A_ receptor associated protein, appears to be crucial for the targeting of the receptor complex in the membrane (Nymann-Andersen et al., 2002). Thus after epileptic seizures it is rapidly internalized but subsequently also rapidly re-synthetized and inserted into the membrane (Naylor et al., 2005, Schwarzer et al., 1997, Tsunashima et al., 1997). The observed lag in decline of γ2 mRNA in this study may reflect transient activation of γ2 mRNA expression. Moreover, the increased expression of α4 is reminiscent to its up-regulation in animal models of temporal lobe epilepsy including electrically-induced status epilepticus and kindling (Nishimura et al., 2005, Sperk et al., 2004, Sperk et al., 1998). Since up-regulation was seen especially in the contralateral hemisphere it also reflects the strong impact of TBI to the entire brain. The α4 subunit is mostly extra-synaptically located and participates in tonic inhibition (Chandra et al., 2006, Sieghart and Sperk, 2002). Its overexpression may be compensatory for the reduced presynaptic GABAergic tone (Pavlov et al., 2011) and could be part of a mechanism protecting the contralateral hemisphere from brain damage. Our data indicate that this seems to be even more obvious for the hippocampus where subunits α4 and α5 (extra-synaptically located; Zhang et al., 2007) are up-regulated in the entire hippocampus at the late 4 months interval. Messenger RNA levels of their presumptive “partners” in the receptor complex, β3, γ2 and δ (Sieghart and Sperk, 2002) were found to be close to control levels at 4 months post-TBI.

### 4.2 Level of the GABA_B1_ and GABA_B2_ mRNAs is decreased in the thalamus but not in the hippocampus after lateral FPI

Presynaptic GABA_B_ receptors are involved in the release of GABA (Bowery et al., 2002). Moreover, postsynaptically these receptors activate G protein-coupled, inwardly rectifying potassium (GIRK) channels (Bowery et al., 2002). Recently, it has been revealed that GABA_B_ receptors can also modulate GABA_A_ receptors via a postsynaptic mechanism and thus regulate tonic inhibitory tone (Connelly et al., 2013, Tao et al., 2013). After an initial down-regulation we observed moderate up-regulation of both GABA_B_ receptor subunits in dentate granule cells 24 h after a status epilepticus induced by kainic acid in rats and in patients with TLE (Furtinger et al., 2003a, Furtinger et al., 2003b). Present study was the first one investigating the change in the GABA_B_ receptor mRNA levels after TBI. We found that besides a transient bilateral up-regulation of GABA_B2_ mRNA in the granule cell layer 6 h after TBI and mild decrease in the GABA_B2_ mRNA level at 4 months post-TBI, transcripts of both GABA_B_ receptor subunits did not significantly change in the hippocampus after TBI. In the thalamus, there was a marked ipsilateral decrease of both subunit mRNAs in the most caudal part of the PO/VPM/VPL complex already at 6 h post-TBI. One possibility is that decreased mRNA levels may reflect underlying neuronal damage in investigated areas.

### 4.3 Quantitative PCR array reproduced results from *in situ* hybridization and revealed change in a pattern of GABA_A_ receptor gene expression after lateral FPI

In the present study, both assessments of the gene expression level gave similar results and the findings were further proved by immunohistochemical analysis. In the dentate gyrus and the hippocampus when subunits on the PCR array were analyzed one by one, only few of them reached the level of statistical significance. Interestingly, when we performed unsupervised hierarchical clustering of all subunits in the granule cell layer or thalamus, we found a change in the pattern of the subunit expression as the analysis separated post-TBI animals from controls. This was not the case in the CA1 subfield of the hippocampus proper. For that reason, our analysis suggests that after TBI in the granule cell layer and thalamus, there is a mild long-lasting GABA_A_ receptor channelopathy which is specific to particular brain areas. If these mild changes are causing altered subunit composition affecting the receptor properties remains to be studied in more detail. However, the expression of GABA_A_ receptor subunits seems to be under coordinated control as it is well known that particular subunits are co-expressed and one can suggest that they share regulatory elements for gene expression (Bailey et al., 1999).

### 4.4 Functional implications

Our study shows complex changes in the constitution of GABA_A_ receptors developing in a first step rather fast (hours or a few days) and are compensated in hippocampus but not in the thalamus in the chronic phase. Does this leave a window for intervention with the pathophysiological events after TBI (e.g. neurodegeneration, development of epilepsy) by augmenting inhibitory transmission through GABA_A_ receptors? GABAergic inhibition involves different cellular mechanisms: 1) Phasic inhibition mediated by synaptically located GABA_A_ receptors mostly assembled by α1-, α2-or α3-, either β- and a γ2-subunit. 2) Tonic inhibition through high affinity GABA_A_ receptors located peri- or extrasynaptically and containing α4-, α5-, either β- and a δ-subunit. Interestingly, tonic GABAergic inhibition appears to be preserved or even increased in animal models of TLE (Scimemi et al., 2005, Zhang et al., 2007) although the α5 and δ subunits become widely down-regulated (Drexel et al., 2013, Nishimura et al., 2005, Sperk et al., 1998, Tsunashima et al., 1997). One explanation for this could be a shift of the γ2-subunit from receptors at synaptic sites to receptors located at peri- and extra-synaptic sites (Zhang et al., 2007). There they may assemble together with α4-subunits that are often preserved in the hippocampus of epileptic rats (Drexel et al., 2013, Sperk et al., 1998).

As mentioned above, in contrast to mRNA levels of subunits α1 and α2 (mediating phasic inhibition) which were comparable to control values in the present study, α4 and α5 subunits (mediating tonic inhibition) become up-regulated in the hippocampus at late intervals after TBI. At these time points, levels of their potential partners, subunits β2, γ2 and δ, were unaltered or also up-regulated. Similarly, in the contralateral thalamus, the receptor configurations consisted of up-regulated α4 subunit and unaltered γ2 or δ subunits. Thus, a therapeutic approach targeting preferentially extra-synaptic, α4, α5 and δ containing GABA_A_ receptors may be promising for augmenting inhibition both in the hippocampus and in the thalamus. Endogenously produced neurosteroids are modulating GABA_A_ receptors through δ-subunit whereas classical benzodiazepines augment GABAergic transmission via an interaction with the γ2 subunit. Neurosteroids like allopregnanolone, allotetrahydrodeoxycorticosterone, and androstanediol as well as progesterone and deoxycorticosterone (that are converted to neurosteroids) exert potent anticonvulsant properties in a variety of animal models of epilepsy (for review see Reddy, 2011). Whether neurosteroids have an influence on the pathophysiology of early brain damage and on development of secondary epilepsy after TBI remains to be studied. O'Dell et al. (2000) studied an approach of activating γ2 subunit containing receptors. Authors observed that treatment with diazepam 15 min prior or 15 min after TBI prevented learning deficits in the Morris maze. Since survival was improved only in rats treated with diazepam prior to TBI these effects seem to be restricted to the very acute phase. It may be reasonable to consider also a combination therapy or stepwise therapy with diazepam and neurosteroids. Because of changes in the expression of chloride transporters NKCC1 and KCC2 in epilepsy the chloride gradient over the neuronal membrane may be altered and may result in an excitatory instead of an inhibitory action of GABA at GABA_A_ receptors (Huberfeld et al., 2007, Rivera et al., 2004). A similar mechanism has also been suggested for TBI (Bonislawski et al., 2007). Thus inhibiting NKCC1 (the chloride transporter thought to transport chloride into the cell) with the selective inhibitor bumetanide results in anticonvulsive actions in some models (Ben-Ari and Tyzio, 2011) and may be even disease modifying in pilocarpine treated animals. Interestingly, phenobarbital acting through GABA_A_ receptors seems to have synergistic effects (Brandt et al., 2010).

## 5 Conclusion

We investigated the time course of mRNA levels of major GABA_A_ and GABA_B_ receptor subunits in hippocampus and thalamus after TBI in the rat. Most GABA_A_ receptor subunits were transiently down-regulated bilaterally in the hippocampus in acute time points. In contrast, in the thalamus mRNA concentrations of all GABA_A_ receptor subunits investigated become rapidly but irreversibly reduced. These thalamic changes were, however, restricted to the ipsilateral side. Whereas the changes in the thalamus may be partly due to the tissue damage, those in the hippocampus seem to reflect TBI related changes in the gene expression. Noteworthy, delayed decreases in γ2 and δ subunit ipsilaterally and increased subunit α4 mRNA levels contralaterally indicate compensatory changes on the gene expression of the GABA_A_ receptor subunits. In addition, bioinformatics analysis of the RT-PCR array suggested mild GABA_A_ receptor channelopathy, particularly in the granule cell layer. Finally, we suggest augmentation of GABAergic transmission with neurosteroids acting through δ-containing receptors as possible strategy.
